# Preclinical Evaluation of UDCA-Containing Oral Formulation in Mice for the Treatment of Wet Age-Related Macular Degeneration

**DOI:** 10.3390/pharmaceutics11110561

**Published:** 2019-10-29

**Authors:** Pooja Maharjan, Daseul Kim, Minki Jin, Hwi Jin Ko, Yeong Ho Song, Yoonjin Lee, Byul-Nim Ahn, Si-Kyung Kim, Yujin Lee, Meong Cheol Shin, Kyoung Ah Min, JaeWook Yang

**Affiliations:** 1 College of Pharmacy and Inje Institute of Pharmaceutical Sciences and Research, Inje University, 197 Injero, Gimhae, Gyeongnam 50834, Korea; maharjan.pooja47@gmail.com (P.M.); k6365d@naver.com (D.K.); jmk2720@nate.com (M.J.); 2 Research Institute, Yoo’s Biopharm Inc., 96 Gamasan-ro, Geumcheon-gu, Seoul 08501, Korea; hwijinko@yoosbiopharm.com (H.J.K.); yhssong@yoosbiopharm.com (Y.H.S.); 3 T2B Infrastructure Center for Ocular Diseases, Inje University Busan Paik Hospital, 75 Bokjiro, Busanjin-gu, Busan 47392, Koreaicetwig@naver.com (B.-N.A.); hush222@naver.com (S.-K.K.); jin49331@nate.com (Y.L.); 4 College of Pharmacy and Research Institute of Pharmaceutical Sciences, Gyeongsang National University, 501 Jinju Daero, Jinju, Gyeongnam 52828, Korea; shinmc@gnu.ac.kr; 5 Department of Ophthalmology, College of Medicine, Inje University, 75 Bokjiro, Busanjin-gu, Busan 47392, Korea

**Keywords:** ocular treatment, ursodeoxycholic acid, tauroursodeoxycholic acid, bile acid, age-related macular degeneration, choroidal neovascularization

## Abstract

As a posterior ocular disease, wet age-related macular degeneration (WAMD) has been known to be related to vision loss, accompanying ocular complications. The intravitreous injection of VEGF antibodies has been reported to be an effective treatment to relieve symptoms of WAMD. However, the limitations of this treatment are high costs and invasiveness. For this reason, oral delivery route can be considered as a cost-effective way and the safest method to deliver drug molecules to the eyes. Accordingly, ursodeoxycholic acid (UDCA) was included in the oral formulation as the potential substance for the cure of WAMD in the animal model. Various pharmacological activities, such as antioxidant or anti-inflammatory effects, have been reported for UDCA and recent reports support the effects of UDCA in ocular treatment. However, due to poor water solubility and low pKa (around 5.0), it has been challenging to formulate aqueous solution of UDCA in the neutral pH range. In the present study, we confirmed the aqueous solubility of the oral UDCA formulation and performed a preclinical study, including pharmacokinetic profiling and WAMD model efficacy study in mice after oral administration of the drug solution. The results demonstrated that the formulation improved bioavailability of UDCA and efficiently delivered UDCA to the eye tissues after oral absorption. UDCA formulation was found to have inhibitory effects of choroidal neovascularization with a functional recovery in mice retinas. Taken together, our results suggest that the oral UDCA formulation could be used as a potent supplement for the cure of WAMD and related retinal diseases.

## 1. Introduction

Age-related macular degeneration (AMD), a leading cause of central vision in individuals aged over 50 years and older, is a disease that generally affects the retinal pigment epithelium (RPE), Bruch’s membrane, and choriocapillaries of the eyes [1,2]. AMD is known to result in severe visual loss due to the development of choroidal neovascularization (CNV) along with related manifestations, such as subretinal hemorrhage, RPE detachment, and fibrovascular disciform scarring, also known as wet or exudative AMD [3]. Among various subtypes of AMD, wet AMD (WAMD) represents only 10–15%; however, over 80% cases of severe visual loss are caused by the wet AMD [4]. Although the pathogenesis of WAMD is poorly understood, it is believed that the hallmark of the exudative AMD is the aging of retina along with apoptosis, inflammation, and angiogenesis [5]. An effective modality to relieve the symptoms of WAMD through inhibiting the action of VEGF in propagating the angiogenesis in retinal endothelial cells has been the intravitreous injection of VEGF antibodies [6,7,8]. However, due to the high costs and invasiveness of this injection, this treatment is not always affordable.

In the pharmacological aspects, the ursodeoxycholic acid (UDCA; 3α,7β-dihydroxy-5β-cholanoic acid), a type of the bile acids, can be a potent agent for the treatment of ocular diseases such as WAMD. For centuries, bile acids, specifically UDCA, have been used in traditional oriental medicine for the treatment of various diseases, including treating inflammatory liver diseases, convulsion and epileptic seizures, renal-lithiasis, cholelithiasis, shrinking tumors, as well as to improve vision [9,10]. To date, UDCA is the only drug approved by the US Food and Drug Administration for the treatment of primary biliary cirrhosis (PBC) [11,12]. UDCA is also known to possess anti-inflammatory activity, as it can increase the expression of neutrophil/monocyte marker- S100A8, directly involved in immune regulation [13]. Moreover, UDCA is also considered as an active agent with antiapoptotic and cytoprotectant activity, because it can prevent cytochrome *c* release, caspase activation and cleavage of poly (ADP-ribose) polymerase. In addition to the aforementioned mechanism, UDCA has been reported to help inhibit apoptosis by decreasing apoptosis inducing molecules in the mitochondria and directly inhibiting ROS production [12,14]. Moreover, previous studies demonstrated that UDCA activates nuclear translocation to modulate the E2F-1/p53/Bax pathway, thereby resulting in antiapoptotic effects [15]. Therefore, with the aforementioned anti-inflammatory, anti-oxidant, anti-apoptotic, cytoprotectant, and anti-angiogenesis-related functions, UDCA could be applicable to treat ocular diseases and, more specifically, age-related macular degeneration.

However, the anatomical structures of the eyes (i.e., retinal location in the posterior segment of the eyes) along with tear dilution eventually create challenges in reaching therapeutic drug concentration in the retinal choroidal regions via ophthalmic drug administration [16]. Therefore, UDCA in the oral formulation can be a potential form of administration to treat ocular diseases. In the previous study, Woo et al. demonstrated that UDCA and its derivative, TUDCA (tauroursodeoxycholic acid), when intraperitoneally injected to the laser-treated rat model, could effectively suppress early VEGF elevation in the retina that inhibits the upregulation of VEGF and eventually results in the reduction in CNV size and vascularity, suggesting the possibility of treating choroidal neovascularization [4]. Despite these effects, however, UDCA has not been developed as an effective oral formulation for the treatment of eye diseases. The disadvantage of UDCA is that, due to poor water solubility and low pKa value (around 5.0), it is challenging to develop a physically stable formulation in the aqueous solution form in the neutral pH range [17,18]. Thus far, oral UDCA formulations as capsules, tablets, or suspensions showed a very low solubility in water and low absorption in the systemic circulation after oral dosing at pharmacological doses (10–15 mg/kg/day) [19,20].

Therefore, in the present study, we confirmed the aqueous solubility of the oral formulation of UDCA and further performed a preclinical study, including pharmacokinetic profiling along with efficacy studies in the mice model. To this end, YSB201 (aqueous solution of UDCA at 25 mg/mL) was used as an oral dosage form. The present study is the first attempt to evaluate the oral bioavailability of the YSB201 formulation in blood and eyes, as well as to study suppression effects of choroidal neovascularization in the WAMD animal model.

## 2. Materials and Methods

### 2.1. Materials

Ammonium acetate, potassium hydroxide (KOH), potassium phosphate monobasic (KH_2_PO_4_), β-NAD (nicotinamide adenine dinucleotide hydrate), EDTA disodium salt dehydrate (EDTA-2Na), 2-mercaptoethanol, and absolute ethanol were purchased from Sigma Aldrich Co. (St. Louis, MO, USA). Acetonitrile and methanol in HPLC grade were obtained from the Burdick-Jackson co. (Muskegon, MI, USA). Standard compounds such as Glycoursodeoxycholic acid (GUDCA), Tauroursodeoxycholic acid (TUDCA), ursodeoxycholic acid (UDCA), Glycocholic acid hydrate (GCA), Taurocholic acid sodium salt hydrate (TCA), Cholic acid (CA), Glycochenodeoxycholic acid (GCDCA), Taurochenodeoxycholic acid (TCDCA), Glycodeoxycholic acid (GDCA), Taurodeoxycholic acid (TDCA), Chenodeoxycholic acid (CDCA), Deoxycholic acid (DCA), Taurolithocholic acid (TLCA), Lithocholic acid (LCA), and 5β-Pregnane-3α,17α,20α-triol (internal standard) were purchased from Sigma Aldrich Co. (St. Louis, MO, USA). Glycolithocholic acid sodium salt (GLCA) was obtained from the Calbiochem (San Diego, CA, USA).

### 2.2. Animal Care

For the pharmacokinetic experiments, we purchased six-week-old female C57BL/6 mice weighing 16–18 g from Koatech (Pyeongtaek, Republic of Korea). For the drug efficacy studies, eight-week-old female C57BL/6 mice were purchased from Orient Bio (Seongnam, Korea). After 6–7 days of acclimatization, the mice were used for the experiments. Animal care and all experiments were performed in accordance with the guidelines for animal experiments of Indang Biomedical Research Center of Inje University Busan Paik Hospital, with the approval of the Institutional Animal Care and Use Committee (No.; IJUBPH_2016-001-02 for pharmacokinetic studies, 3 October 2017; 2016-T2B-003-03 for the efficacy studies, 8 January 2016). The mice were housed under controlled environment at the temperature of 19–25 °C, light of 150–300 Lux, and humidity of 40–60%. The animals were fed with Laboratory Rodent Chow W (SAFE Complete Care Competence, France) *ad libitum*.

### 2.3. Establishment of High-Performance Liquid Chromatography (HPLC) Setup

**Figure 1:**
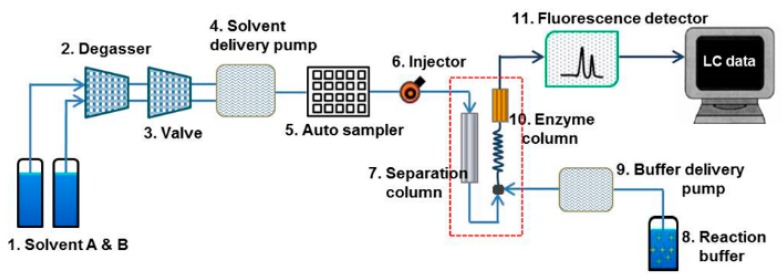
High-Performance Liquid Chromatography (HPLC) setup with a fluorescence detector (excitation: 345 nm, emission: 470 nm) for the analysis of bile acids. The Schematic diagram of bile acids analysis system shows the Waters^®^ 2695 Alliance HPLC system equipped with analysis components (**1** through **11**). The flow direction through column sets (**7.** Separation column, **10.** Enzyme reaction column) is indicated by arrows. The flow from the component **7** (separation column) is merged with flow from the component **9** (reagent pump), and then the mixtures of mobile solvents with the separated eluents and reagent solution comes through the reaction coil into the Enzymepakcolumn (component **10**). Finally, the flow from the Enzyme column goes into the fluorescence detector (component **11**).

A Waters^®^ Alliance HPLC system (Waters, Milford, MA, USA) with a fluorescent detector was used for the sample analysis throughout. The entire HPLC system was controlled by the Empower 3 software. All chromatographic separations were performed with a JASCO BilePak II 5 µm C_18_ column (250 mm × 4.6 mm ID) equipped with an Aspentis C18 guard column (Sigma, MP, USA). EnzymePak 3α-HSD column (35 mm × 4.6 mm ID) (JASCO, Tokyo, Japan) was linked for the LC pump with the enzyme reaction buffer. JASCO’s biliary acid analysis system of Japan was used for the analysis of blood and biological samples of mice containing UDCA component and its associated bile acid-based substance [21]. A Waters Alliance HPLC system was constructed to quantitatively analyze the concentration of bile acid using a fluorescence detector (Figure 1; HPLC system setup). The mobile solvent A consisted of acetonitrile (ACN):methanol (MeOH):30 mM ammonium acetate (30:30:40) while the mobile solvent B was prepared as ACN:MeOH:30 mM ammonium acetate (20:20:60). The enzyme reaction buffer solution contained 0.3 mM β-NAD, 10 mM KH_2_PO_4_, 1 mM EDTA-2Na, and 0.05% 2-mercaptoethanol, with final adjustment at pH 7.8 by potassium hydroxide (KOH).

To determine bile acid components, mobile solvents were flown at the flow rate of 1 mL/min by the gradient method in 100% solvent B for the first 32 min, 100% solvent A from 32 min to 60 min, 100% solvent B from 60 min to 65 min. The standard solution (10 µL) injected into HPLC was separated into each bile acid by gradient elution from BilePak II column (“7” in Figure 1) by the mobile solvents A and B. In the next step, the solution was mixed in the reaction coil with the enzyme reaction solution containing β-NAD. When the enzyme entered the EnzymePak 3α-HSD column (“10” in Figure 1) with 3α-HSD (hydroxy steroid dehydrogenase), the signal of each compound was measured in the fluorescence detector (“11” in Figure 1) (excitation 345 nm/emission 470 nm) due to the dihydronicotinamide adenine dinucleotide (NADH) produced by the enzyme reaction in the column. This post-column reaction method was used to separate and detect 15 bile compounds and internal standard in the standard and sample solutions.

### 2.4. Preparation of Bile Standard Solutions for Calibration Curves

The following 15 types of bile acids and metabolites, including primary and secondary bile acids and glycine and taurine-conjugated forms, were used in the present study: Glycoursodeoxycholic acid (GUDCA), Tauroursodeoxycholic acid (TUDCA), Ursodeoxycholic acid (UDCA), Glycocholic acid hydrate (GCA), Taurocholic acid sodium salt hydrate (TCA), Cholic acid (CA), Glycochenodeoxycholic acid (GCDCA), Taurochenodeoxycholic acid (TCDCA), Glycodeoxycholic acid (GDCA), Taurodeoxycholic acid (TDCA), Chenodeoxycholic acid (CDCA), Deoxycholic acid (DCA), Glycolithocholic acid sodium salt (GLCA), Taurolithocholic acid (TLCA) and Lithocholic acid (LCA). Each of these 15 standard bile acids was weighed and dissolved in methanol to make 3 mM stock solution and then stored at −80 °C. 5β-Pregnane-3α, 17α, 20α-triol as the internal standard (IS) was dissolved in methanol at 1 mM and stored at −80 °C.

Plasma and eyes were pooled from the untreated mice. Eye tissues were grinded by IKA^®^ Ultra-turrax homogenizer with the addition of the cell lysis solution (iNtRON biotechnology, Seoul, Korea) in milliQ water (1:4). To remove the endogenous bile compounds in the tissues, the eyes or plasma of the mice were incubated with 100 mg/mL activated charcoal solution for 90 min at room temperature [22]. After centrifugation at 3500 rpm for 10 min, only the supernatant was collected and used further for construction of standard solution. In order to construct the calibration standard set with the concentration range (0.2, 0.5, 1, 2, 10, 50, 100 μM), the mixture of 15 bile standard compounds was spiked into each type of biological matrix (plasma or eyes), and then IS was finally added into the standard solution with the final IS concentration of 10 μM. The calibration curve was prepared from the linear regression method based on the relative ratio of peak area of the bile acid compound to IS peak area within the concentration range.

### 2.5. Biomatrix Sample Extraction

To extract the tissue solution, the protein precipitation technique with ice-cold ethanol was used. 500 μL of the homogenized supernatant of eyes or plasma with 50 μL of IS spiked were mixed with 2.5 mL of ice-cold absolute ethanol. After heating in a water bath for 1 min and centrifugation at 3500 rpm for 5 min, supernatant was obtained. The residue was used for further extraction procedure. After the remaining residue was shaken with 1 mL of cold ethanol, the mixture was incubated at 85 °C for 1 min. After centrifugation at 3500 rpm for 5 min, the supernatant was obtained. As the final step, the remaining residue was shaken with 1 mL of cold ethanol, and the mixture was incubated at 85 °C for 1 min. Finally, after centrifugation at 3500 rpm for 5 min, the supernatant was obtained, and a total of three supernatant liquid fractions were collected. Supernatants from 3 extraction steps were evaporated using an EYELA centrifugal vacuum concentrator (CVE-2200, Tokyo, Japan) at 1400 rpm for 2 h. The residue was reconstituted with 500 μL of methanol, filtered through a 0.45 μm PTFE syringe filter, and analyzed with 10 μL injection of sample volume under the HPLC equipment conditions specified above. Extraction recoveries (%) were calculated for each quality control (QC) concentration (0.5, 1, 10, 50, or 100 μM) in each biomatrix (eyes or plasma) based on the peak area ratio of analytes and IS in the extracted solution, as compared to the corresponding peak area ratio in the unextracted solution.

### 2.6. Fluorescent HPLC Method Validation

For further application of the HPLC analysis method using the fluorescent detector in samples, method validation for each calibration curve in biomatrix type (eyes or plasma) of mice has to be performed. Accordingly, five replicates of each QC point of bile acid concentrations in plasma were analyzed to determine the intra-day precision. The intra-day precision was calculated from the relative standard deviation (RSD, % = standard deviation of peak area ratio/mean of peak area ratio × 100). Inter-day validation was performed with each QC standard set (0.5–100 μM) on three different days for plasma and the homogenized eye supernatant solution. Inter-day precision was calculated as RSD (%) in intra-day validation, and inter-day accuracy was determined using Equation (1).

(1) $$\mathrm{Accuracy}\;\left(\%\right)\;=\;\frac{\mathrm{Measured}\;\mathrm{concentration}\;\left(\mu M\right)}{\mathrm{Theoretical}\;\mathrm{concentration}\;\left(\mu M\right)}\times 100$$

### 2.7. In Vivo Pharmacokinetic Studies

#### 2.7.1. Drug Administration and Sample Collection

The set of C57BL/6 mice in the control or treatment groups were not fed for 12 h before the experiments. The aqueous solution of YSB201 including UDCA (25 mg/mL) from Yoo’s Biopharm Inc. (Seoul, Korea) was orally administered with the dose of 125 mg/kg to mice in the treatment group once by the disposable syringe with an oral zonde. The oral dose to each mouse was calculated based on the body weight of the animal measured after fasting for 12 h prior to oral administration of YSB201. After 4 h from oral drug administration, the mice were re-fed with a solid diet. At time points including 0, 5, 10, 30 min, and 1, 2, 4, 10, 24, 48, and 72 h after oral administration of YSB201 (125 mg/kg) in C57BL/6 mice, blood and eye tissues were collected from four mice. Blood was collected into heparinized tubes from each mouse and centrifuged at 3000 rpm for 5 min to collect plasma. Both eyes were harvested from each mouse and stored at −80 °C until further analysis.

#### 2.7.2. Pharmacokinetic Analysis

Drug components including bile acid metabolites were extracted from the tissue using an organic solvent (protein precipitation method with ethanol) [23]. Homogenized supernatant of eyes or plasma (500 μL) were mixed with 50 μL of IS, subjected to the extraction process using ice-cold absolute ethanol, and finally reconstituted in 500 μL of methanol. The peak areas of UDCA and its metabolites in the extracted mouse plasma or eye tissues in treatment group were quantitated using the established HPLC analysis method with enzyme reaction and fluorescence detection. The concentration of each bile acid component in each biomatrix sample solution was calculated using the calibration curves based on the plasma or eye tissue standard solution. The results of the analysis were applied to a pharmacokinetic analysis program Pharsight WinNonlin 7.0 (Certara, NJ, USA) to calculate the pharmacokinetic parameters after oral administration of the test agent and to confirm the pharmacokinetic profile of the YSB201 formulation. The following values were evaluated: maximum plasma concentration (C_max_); time to reach the C_max_ (T_max_); area under the plasma concentration-time curve from 0 to 48 h (AUC_0–48h_); elimination rate constant (k); half-life (t_1/2_); and mean residence time (MRT) were calculated using the noncompartmental analysis.

### 2.8. In Vivo Ocular Efficacy Study in Mice

#### 2.8.1. Drug Administration and Sample Collection

The C57BL/6 female mice weighing 16–18 g were used to develop a laser-induced choroidal neovascularization (CNV) mouse model to test YSB201 agent’s efficacy in the treatment of age-related macular degeneration (AMD). A total of 24 mice were classified into four groups: Group 1 (control group without a laser injury and drug treatment); Group 2 (mice with a laser injury and only vehicle (olive oil) administration); Group 3 (mice with a laser injury and administration of 125 mg/kg YSB201); and Group 4 (mice with a laser injury and administration of 250 mg/kg YSB201). In each group, six mice were tested. In Groups 2, 3, 4, vehicle or YSB201 at different concentrations (125 mg/kg vs. 250 mg/kg) was orally administered to the mice (*n* = 6) in each group once a day between 11:00 a.m. and 2:00 p.m. from 10 days before a laser injury. After a laser injury, the mice were orally administered once a day with vehicle or YSB201 at 125 or 250 mg/kg for 10 days between 11:00 am and 2:00 pm. Finally, to collect the samples for further analysis, the mice in the control and each treatment group were euthanized on day 15 after the date of the laser injury.

#### 2.8.2. Laser Induced Choroidal Neovascularization

The C57BL/6 mice were anesthetized with ketamine hydrochloride (30 mg/kg, body weight, Huons, Jacheon, Republic of Korea) and xylazine hydrochloride (2.5 mg/kg body weight, Bayer Korea Ltd., Seoul, Republic of Korea), and the pupils were dilated with topical drops of 0.5% proparacaine hydrochloride (Tropherine Eye Drops; Hanmi Pharmaceutical Co., Ltd., Seoul, Republic of Korea). In order to generate choroidal neovascularization (CNV), laser photocoagulation was induced (532 nm, 200 mW, 70 ms, 50 µm; Micron IV, Phoenix Research Laboratories, CA, USA). Thirteen days after laser photocoagulation, the fundus fluorescein angiography (FFA) was performed using an intraperitoneal injection of 1% fluorescein (Sigma, MO, USA). The fluorescein leakages were represented by the corrected total fluorescence (CTF) calibrated with the ImageJ software (National Institutes of Health, MD, USA) using the Equation (2).

CTF = (Integrated Density) − [(Area of selected lesion) × (Mean fluorescence of background readings)](2)

Optical coherence tomography (OCT) was performed using the Micron IV imaging system (phoenix Research Labs, CA, USA). The OCT images were captured per each lesion to allow calculation of CNV lesion size. For the quantification of CNV area, the diameters were manually drawn and measured with the ImageJ software (National Institute of Health, Bethesda, MD, USA).

#### 2.8.3. Western Blot Analysis

Western blots were performed to evaluate the vascular endothelial growth factor (VEGF) expression levels in mice eyes in control vs. each treatment group after the CNV induction. At 15 days after laser injury, the mice were euthanized and the choroid and retinal layers were separated by removing the sclera, cornea and lens from the eyeballs. The choroidal and retinal tissues were washed twice with PBS, and proteins were extracted by homogenizing the tissue with the protein extraction solution, Pro-PREP (Intron Biotechnology, Gyeonggi-do, Korea). Two eyes from each mouse were considered as one sample. Protein concentrations were determined using the BCA protein assay kit (Thermo scientific, WI, USA), and 20 μg of total protein were analyzed by western blotting. Proteins were separated by sodium dodecyl sulfate-polyacrylamide gel electrophoresis (SDS-PAGE) and transferred onto a nitrocellulose membrane. After blocking with 5% nonfat dried milk in Tris-buffered saline with Tween 20 (TBS-T) for 1 h, the membrane was incubated with a specific primary antibody overnight at 4 °C; rabbit polyclonal anti-VEGFA (abcam, Cambridge, MA, USA) diluted at 1:1000 in TBS-T, rabbit polyclonal beta-actin (abcam, Cambridge, MA, USA) diluted at 1:5000 in TBS-T. The blots were then horseradish peroxidase-conjugated secondary goat anti-rabbit antibody in TBS-T buffer containing 3% nonfat dried milk for 1h at room temperature, and immune complexes were detected using a ProNA ECL Ottimo detection kit (TransLab, Daejeon, Korea). The cooled CCD camera system Fusion FX Image acquisition system (Vilber Lourmat, Torcy, France) (Vilber Lourmat, Marne La Vallee, France) was used to detect band intensities.

#### 2.8.4. Electroretinography Measurement

Electroretinography (ERG) recordings were obtained using Micron Ganzfeld ERG (Phoenix Research Lab). The analysis was conducted with the anesthetized mice after 24 h dark-adaptation on day 14 after laser photocoagulation. The electrodes were placed on the skin, tail, and cornea for electroretinography. The response was measured by stimulating the retina with a single white light (0.8 cd·s/m^2^ of flash intensity), and the B wave amplitude was analyzed by LabScribeERG software (Phoenix Research Laboratories, CA, USA).

### 2.9. Data Analysis

Data analyses were performed using Microsoft Excel 2010 and GraphPad Prism 5.03 (GraphPad Software; LaJolla, CA, USA). As a statistical analysis, one-way ANOVA with the significance level of 0.05 was used. Tukey’s multiple comparison test was performed as a post test for one-way analysis of variance.

## 3. Results

### 3.1. Assessment of Fluorescent HPLC Method Validation

#### 3.1.1. Specificity and Calibration Linearity

**Figure 2:**
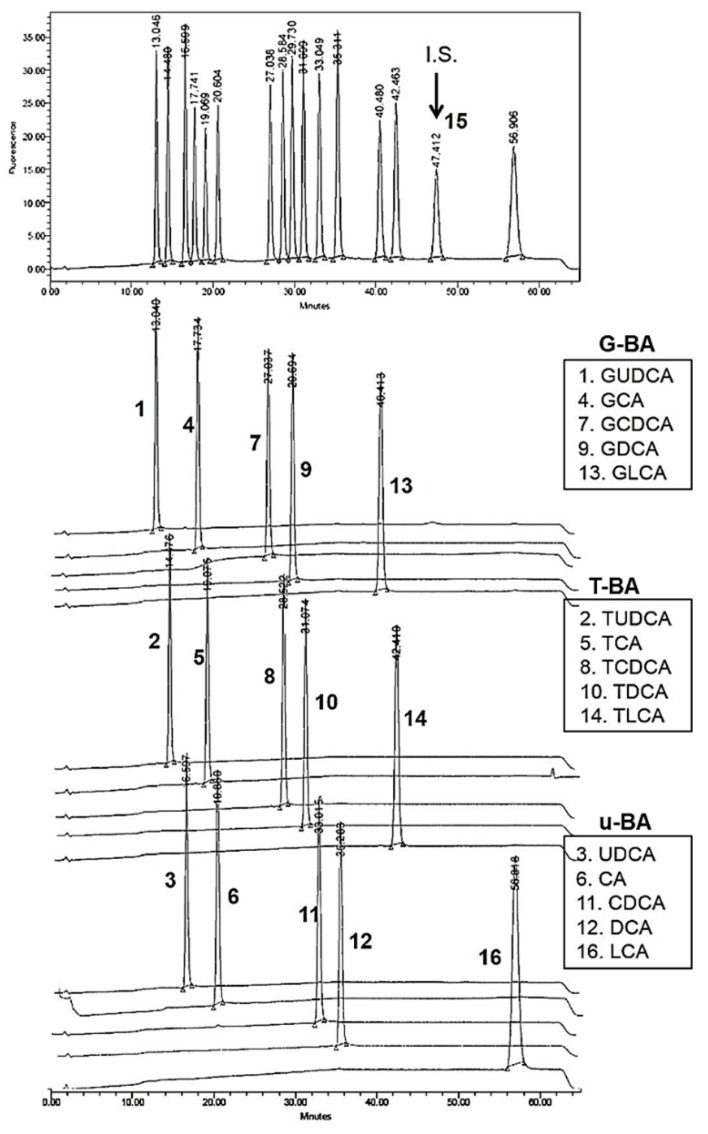
HPLC chromatogram of bile acid standard solution with 5β-Pregnane-3α, 17α, 20α-triol (IS) in methanol. Bile acid standards include 15 types of bile acids such as glycine-conjugated bile acids (G-BA: #1, #4, #7, #9, #13), taurine-conjugated bile acids (T-BA: #2, #5, #8, #10, #14), and unconjugated bile acids (u-BA: #3, #6, #11, #12, #16). Below the chromatogram of standard mixtures and IS compound (#15), each chromatogram obtained from the HPLC analysis separately performed with each bile acid compound was displayed with the specific retention time (min).

Calibration solution containing a standard compound such as UDCA and its glycine- or taurine-conjugated or unconjugated metabolites was prepared in methanol with 10 μM of each compound as the final concentration. In the chromatogram of the bile acids standard solution, the peaks of respective components showed up separately from each other at each retention time (Figure 2). The retention times averaged over five different batches were as follows: 12.3 ± 0.37 min for compound #1 (GDCA); 13.7 ± 0.38 min for compound #2 (TUDCA); 15.8 ± 0.37 min for compound #3 (UDCA); 16.9 ± 0.41 min for compound #4 (GCA); 18.2 ± 0.41 min for compound #5 (TCA); 19.6 ± 0.39 min for compound #6 (CA); 26.0 ± 0.35 min for compound #7 (GCDCA); 27.5 ± 0.33 min for compound #8 (TCDCA); 28.6 ± 0.32 min for compound #9 (GDCA); 30.0 ± 0.30 min for compound #10 (TDCA); 31.9 ± 0.29 min for compound #11 (CDCA); 34.1 ± 0.27 min for compound #12 (DCA); 39.0 ± 0.29 min for compound #13 (GLCA); 40.8 ± 0.32 min for compound #14 (TLCA); and 54.4 ± 0.51 min for compound #16 (LCA). The #15 peak as the internal standard (IS, 10 μM) was shown at 46.0 ± 0.34 min.

**Figure 3:**
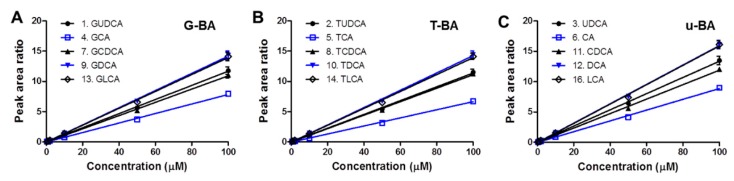
Calibration curve linearity of the standard bile acids. Calibration lines within the concentration range of 0.2–100 μM are displayed for (**A**) glycine-conjugated bile acids (G-BA compound No. 1, 4, 7, 9, 13), (**B**) taurine-conjugated bile acids (T-BA compound No. 2, 5, 8, 10, 14), and (**C**) unconjugated bile acids (u-BA compound No. 3, 6, 11, 12, 16).

In order to confirm the linearity of the calibration curve, bile acid standard solution was prepared with the concentration range (0.2, 0.5, 1, 2, 5, 10, 50 and 100 μM) of each bile acid standard compound in methanol (Figure 3A–C). With 10 μM internal standard (IS) spiked in the solution, the calibration solution was injected into HPLC and analyzed by the fluorescent LC set-up. Peak areas of compounds (#1–#16) at each peak retention time were analyzed in the chromatograms of the bile acid standard solution within the concentration range (0.2–100 μM). Using the analysis of each chromatogram, the peak area ratio was obtained by dividing the peak area value of the chromatogram of each of the bile acids (#1–#14 & #16) by the peak area of IS (#15). The calibration curve of bile acid standard solution in methanol with IS spiked was drawn for each bile acid using peak area ratios vs. compound concentration (Figure 3A–C). The linearity of the calibration curves of glycine-, taurine-conjugated or unconjugated bile acid standard solution (G-BA, T-BA, or u-BA, respectively) were confirmed with the slopes of the calibration curve and the *y*-axis intercept values by using the linear regression method. These calibration curves were used to calculate the concentration of extracted bile components in each blood and eye samples in the mice group.

#### 3.1.2. Validation Results with Quality Control Analysis in Biomatrices

To evaluate the pharmacokinetic profiles in the mice after oral administration of YSB201, in addition to UDCA (the main component of the test product YSB201), conjugated or unconjugated forms of UDCA metabolites should be quantitatively analyzed in the mice plasma or eye samples of the test group. Therefore, before analyzing the biological samples of the mouse group of the test group treated with the test agent, the HPLC analysis method and also extraction procedures were validated using the standard mixtures of 15 bile acid type compounds in the blank plasma or eye homogenates. In order to test the reproducible analytical condition with the fluorescent HPLC set up, the method was defined by examining both intra- and inter-day variance as RSD values (%), which are recommended to be within the range of 80–120%. The inter-day validations were performed with the standard mixtures of 15 types of bile acids on different 3 days in the blank plasma or eye homogenates of mice and the intra-day validations were conducted with the repeated HPLC analysis within a day of analysis. As mentioned in the method section, the relative standard deviation (RSD) was calculated as % by dividing the standard deviation values of peak area ratio by the average peak area ratios based on the chromatogram results. Validation results with intra- and inter-day RSD values in plasma or eyes for 15 bile acid types (G-BAs, T-BAs, or u-BAs) are reported as precision results in Table 1, Table 2 and Table 3.

The accuracy of the HPLC assay for UDCA and other bile acids at the concentration higher than the lower limit of quantification concentration (LOQ) showed that this concentration deviated from the true concentration by only 15% as calculated from the linear regression equations of the calibration curves (Table 1, Table 2 and Table 3). An extraction method showing recovery of 90% or more for the concentration ranges of 15 types of bile acids was also established using the organic solvent (data not shown).

### 3.2. Aqueous Solubility and Drug Content of Oral UDCA Formulation

**Figure 4:**
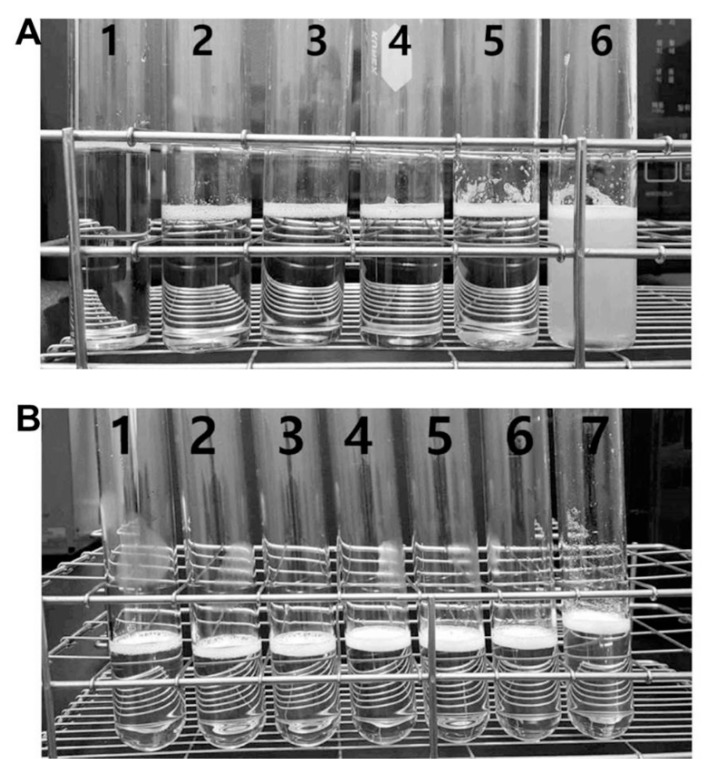
Physical examination of the aqueous YSB201 formulation under various pH condition. (**A**) For the formulation with the weight ratio of ursodeoxycholic acid (UDCA) to maltodextrin (1:15), the solution in tubes is displayed with numbers at different pH conditions; pH 9.5 (**1**, **clear**), pH 8.9 (**2**, **clear**), pH 7.9 (**3**, **clear**), pH 7.1 (**4**, **clear**), pH 6.0 (**5**, **clear**), pH 5.5 (**6**, **precipitated**). (**B**) With the weight ratio of UDCA to maltodextrin (1:30), the solution in tubes is displayed with numbers at different pH conditions; pH 9.0 (**1**, **clear**), pH 8.0 (**2**, **clear**), pH 7.0 (**3**, **clear**), pH 6.0 (**4**, **clear**), pH 5.1 (**5**, **clear**), pH 4.1 (**6**, **clear**), pH 2.9 (**7**, **clear**).

The aqueous solubility of the YSB201 formulation with different weight ratios of UDCA to maltodextrin was evaluated under various pH conditions in the solution. As shown in Figure 4, the formulation with the weight ratio of UDCA to maltodextrin (1:15) showed precipitation in the solution at pH 5.5. Under the higher pH condition than 5.5, up to pH 9.5, the solution showed little precipitation. However, with the weight ratio of UDCA to maltodextrin (1:30), the formulation solution in tubes showed stability with a clear solution at different pH condition (pH 2.9–9.0).

**Figure 5:**
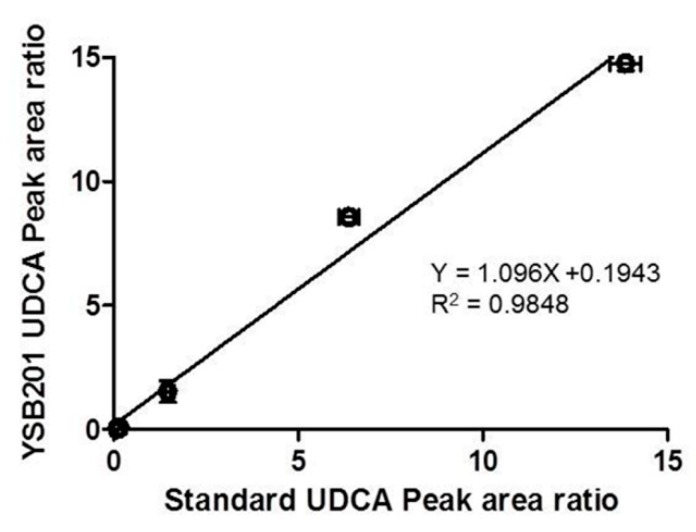
Assessment of purity of the YSB201 formulation by the standard UDCA. The UDCA peak area ratios compared with IS peak area in the diluted YSB201 agent were compared with the UDCA peak area ratio values of the standard UDCA.

For the YSB201 formulation with the weight ratio of UDCA to maltodextrin (1:30) at pH 7.0, the UDCA content in the solution was analyzed by the established fluorescent HPLC method. The UDCA peak of the standard UDCA reagent was compared with the UDCA peak of the YSB201 solution. The purity of the YSB201 formulation was confirmed by the comparison of the UDCA peak area ratio to IS peak area of the diluted YSB201 agent and the standard UDCA reagent (Figure 5).

### 3.3. Pharmacokinetics of UDCA and Metabolites in Plasma and Eyes

**Figure 6:**
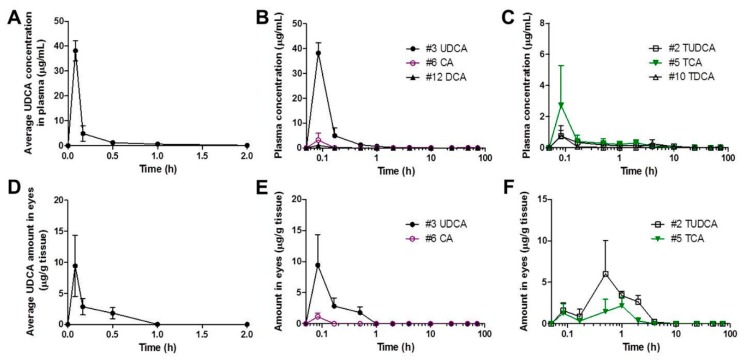
Average concentration of UDCA and its metabolic derivatives in the plasma or eyes after the oral administration of YSB201 (125 mg/kg). (**A**) Average UDCA concentration (μg/mL) in plasma of the treatment group mice up to 2 h after YSB201 oral administration (*n* = 3). Average concentrations of (**B**) unconjugated bile acids or (**C**) Taurine-conjugated bile acids in the plasma of the treatment group are displayed with the time in the logarithmic *x*-axis (0–72 h). Mean ± SEM (*n* = 3). (**D**) Average UDCA amount (μg/g of tissues) in eye tissues of the treatment group mice up to 2 h after YSB201 oral administration (*n* = 3). Average concentrations of (**E**) unconjugated bile acids or (**F**) Taurine-conjugated bile acids in the treatment group are displayed with the time in the logarithmic *x*-axis (0–72 h). The amounts of bile acids in the treatment group mice were calculated by subtracting the bile acid background signals of control mice (*n* = 3) on the same diet as that of the treatment group mice (*n* = 3).

After oral administration of YSB201 at 125 mg/kg in the mouse model of C57BL/6, plasma samples and eye tissues were collected from mice at each time point (5, 10, 30 min, and 1, 2, 4, 10, 24, 48, and 72 h). As drug components of YSB201, UDCA and its metabolic derivatives were extracted from tissue samples using the established extraction method with the organic solvent. In order to evaluate the pharmacokinetic trend of UDCA and its metabolites into plasma and eyes after oral administration of YSB201, the HPLC analysis method using enzyme reaction and fluorescence detection was applied to quantitate concentrations of UDCA and its metabolites extracted from mice plasma or tissue. The average UDCA concentration vs. time profiles in mice plasma or eyes (*n* = 3) are shown in Figure 6A,D. The calculated pharmacokinetic parameters of UDCA in mice plasma or eyes are listed in Table 4. According to the results, the concentration of UDCA in the blood of the mice reached its maximum plasma concentration (38.18 ± 7.05 (SD; standard deviation)) μg/mL within 5 min after oral administration of the YSB201, and showed an average half-life of about 1.12 h (±0.15). The drug UDCA tended to disappear from the blood between 10–24 h after the administration, and the UDCA concentration in plasma (*n* = 3) was below the quantitative limit (<0.2 μM) after 24 h post dosing. As shown in Figure 6A, UDCA appeared to be quite quickly distributed to tissues from the systemic circulation and disappeared within a short time in the body.

After two eyes of the test group mice were extracted and quantitatively analyzed under the same condition as the blood sample, the highest concentration was reached in the eyes between 5 and 30 min after oral administration, and the UDCA concentrations were not detected in the eyes at 2 h after the administration. Pharmacokinetic modeling of oral medication with the noncompartmental analysis (NCA) was performed to calculate major pharmacokinetic parameters (Table 4). The AUC (i.e., the area under the blood concentration curve) value from 0 h to 48 h was calculated to be equal to the AUC up to 72 h or the infinite time due to completely undetectable concentrations of UDCA in plasma at 48 h after drug administration. For eye samples, the AUC value was calculated as μg/g of eye tissues from the curve of drug concentration vs. time up to 48 h at the same scale of AUC of plasma samples. Overall, variation errors (RSD, %) of pharmacokinetic parameters in mice plasma or eyes were within 20%.

**Figure 7:**
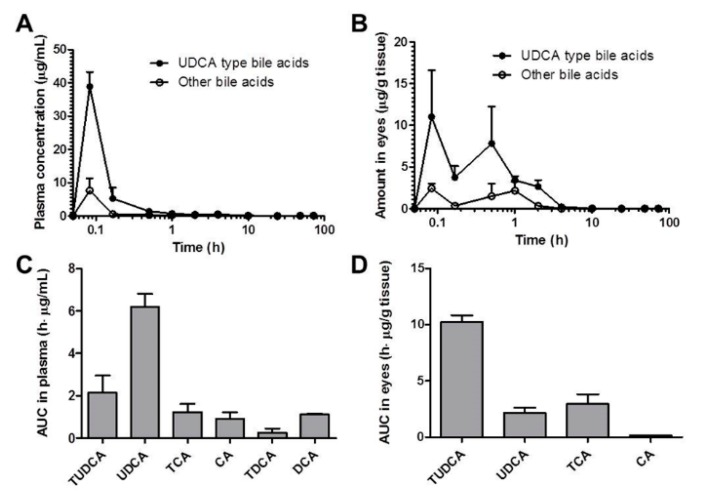
Average concentration and area under the bile concentration-time curve (AUC) of UDCA and other bile acids in plasma (**A**) or eyes (**B**) after the oral administration of YSB201 (125 mg/kg). AUC values (**C**,**D**) were calculated up to 48 h after the drug administration by the noncompartmental analysis of Pharsight^®^ WinNonlin program (Certara L.P., Princeton, NJ, USA).

In Figure 6B,C, the levels of UDCA metabolites in the mice plasma could be seen with the time after YSB201 administration. Figure 6E,F showed the concentration changes of UDCA metabolites in the mice eyes after drug administration. According to the results, major metabolites of UDCA after YSB201 administration appeared to be TUDCA and TCA in plasma and eyes. As unconjugated forms of bile acids, CA and DCA were also found in mice plasma within 1 h after UDCA administration. This could mean that the levels of those unconjugated bile forms may have increased if the higher dose of drug was used. There were no glycine-conjugated forms found in plasma or eyes. Figure 7A,B show the trends of UDCA type bile acids (the sum of levels of UDCA and TUDCA) vs. other types of bile acids with the time after YSB201 administration (125 mg/kg). At 30 min after the administration, in the eyes, the total UDCA-type bile acid levels appeared to be higher than other types of bile acids due to the increased TUDCA. Figure 7C,D show the AUC values of each type of bile acids found in the mice plasma and eyes, indicating that major bile acids were UDCA in plasma and TUDCA in eyes, respectively.

### 3.4. Inhibitory Effects of Choroidal Neovascularization by Oral UDCA Formulation

**Figure 8:**
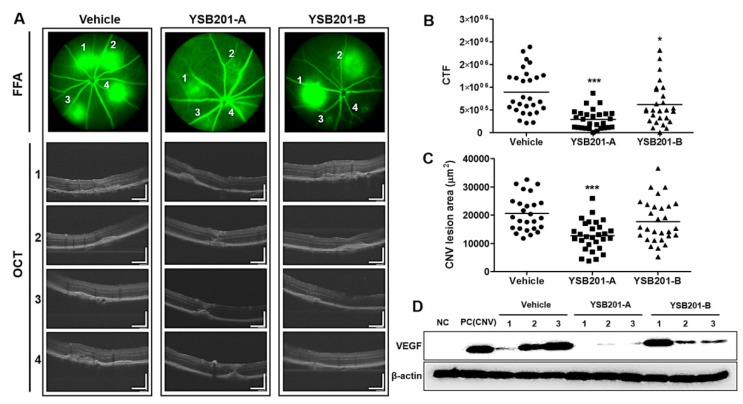
Response examination of the choroidal neovascularization 13 days after CNV induction and oral administration of YSB201. (**A**) Based on representative FFA images of each treatment group, OCT was captured for each CNV lesion (no. 1–4) and displayed with the scale bar (200 μm). The quantification was performed for the FFA images of each group to obtain (**B**) the fluorescein leakage areas (the corrected total fluorescence; CTF). (**C**) CNV lesion areas were assessed using the ImageJ software based on OCT results. The CTF or CNV lesion areas in the YSB201-A (125 mg/kg) or YSB201-B (250 mg/kg)-treated group were noted as compared to that in the vehicle-treated group. * *p* < 0.05, ** *p* < 0.01, *** *p* < 0.001 by 1-way ANOVA test (significance level = 0.05). (**D**) Western blot analysis was performed for the detection of VEGF expression levels in each treatment group (oral administration of YSB201-A or -B after CNV induction) (*n* = 3), as compared with NC (negative control without CNV) and PC (positive control after CNV induction). Expression of β-actin is shown as a loading control.

At 13 days after laser injury, 1% fluorescein solution was intraperitoneally injected to confirm the CNV at the site of laser irradiation and optical coherence tomography (OCT) response was examined for each CNV lesion (Figure 8A). The fluorescein leakages were represented with the corrected total fluorescence (CTF) based on the fundus fluorescein angiography (FFA). Compared to the CTF from the vehicle (928,002 ± 494,908), the CTF values from the 125 mg/kg and 250 mg/kg of YSB201 were 383,227 ± 432,802, and 771,550 ± 736,401, respectively (Figure 8B). Distinct regions of the hemorrhage in FFA images (no. 1–4) in each treatment group were examined by the OCT angiography in Figure 8A. The cross-sectional angiograms visualized CNV location relative to the outer retinal membrane and Bruch’s layer. In result, the regions of the subretinal hemorrhage could be apparently seen in the vehicle group. The CNV lesion areas became smaller to 12,684 ± 5210 or 17,761 ± 7576 by drug treatment (125 mg/kg or 250 mg/kg of YSB201, respectively), compared to the lesion area from the vehicle group (20,553 ± 6205) (Figure 8C). After the oral administration of 125 mg/kg of YSB201 (YSB201-A), laser-induced CNV was significantly reduced in the CTF values and CNV lesion sizes from OCT images when statistically compared with the vehicle. Therefore, oral administration of YSB201 successfully contributed to reducing CNV levels and sizes in the eyes. In addition to the examination of the CNV levels in the mice eyes by imaging, the western blot analysis was performed to evaluate the expression of the vascular endothelial growth factor (VEGF) in the choroid and retina of mice (Figure 8D). The VEGF expression was significantly increased in the positive control group (CNV induction after a laser injury) or in the vehicle mice group, compared with the negative control without a laser injury. Meanwhile, in the mice group administered by 125 mg/kg of YSB201, the expression of VEGF was significantly decreased, suggesting the down-regulation of the VEGF expression in the choroidal and retinal cells by the oral administration of YSB201 formulation.

**Figure 9:**
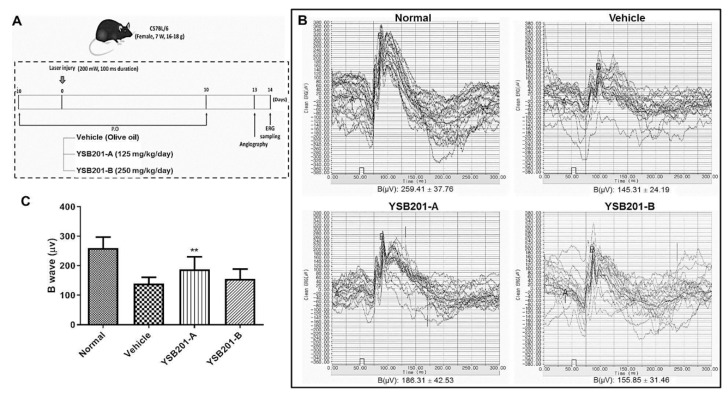
Measurement of the ERG response 14 days after CNV induction and oral administration of YSB201. (**A**) The diagram represents the experimental scheme with CNV model establishment and drug administration schedule. (**B**) As of the representative dark-adapted ERG response, A and B wave amplitudes are shown for the normal mice and treatment group with the vehicle or YSB201-treated group (YSB201-A (125 mg/kg), YSB201-B (250 mg/kg)). (**C**) The B-wave amplitudes of ERG data in the YSB201-A (125 mg/kg) or YSB201-B (250 mg/kg)-treated group are shown with the data in the normal or the vehicle-treated group. * *p* < 0.05, ** *p* < 0.01, *** *p* < 0.001, by 1-way ANOVA test (significance level = 0.05).

To evaluate the effects of YSB201 on retinal function in the CNV-induced mice model, we performed electroretinography (ERG) measurements and compared the CNV-induced mice group treated with YSB201 or vehicle with the group of normal mice without CNV. The amplitude of B wave was 259.4 ± 37.8 μV in the normal group and 140.0 ± 20.7 μV in the vehicle-treated group, which indicated a decline in the retinal function by the CNV and no treatment effect by the vehicle. Compared to the ERG data of the vehicle group, in the treatment group with YSB201 (125 mg/kg), attenuation of B wave amplitudes was significantly inhibited (188.0 ± 42.2), suggesting the recovery of the retinal function in the CNV disease model by oral administration of YSB201 (125 mg/kg) (Figure 9). Figure 9C shows that the inhibition of attenuation of B wave amplitudes by YSB201 (125 mg/kg) was statistically significant, as compared to the amplitudes by the vehicle group (** *p* < 0.01).

## 4. Discussion

In the present study, the aqueous UDCA solution (YSB201) was used as an oral formulation in mice and pharmacokinetic profiles were analyzed along with drug efficacy in retinal choroids of mice eyes. YSB201 (a high concentration of UDCA aqueous solution) developed as an oral dosage form showed an increased water solubility of UDCA to 25 mg/mL. We examined whether this formulation could deliver a therapeutically effective dose of UDCA to the blood and eyes when given orally. Presumably due to the physicochemical properties of UDCA itself, the results of several previous reports suggested that the poor absorption and rapid distribution of UDCA may prevent tissues from attaining sufficiently high concentrations and maintaining therapeutic effects as an in vivo pharmacological agent (i.e., anti-inflammatory or antioxidant efficacy) after the oral delivery [24,25]. However, in other studies, YSB201 solution containing soluble UDCA was demonstrated to possess antioxidant and anti-apoptotic effects [26,27,28]. Considering these pharmacological reports, it is important to obtain pharmacokinetic information from a preclinical study to ascertain the contribution of the parent compound, UDCA, and any of its active metabolic derivatives to the pharmacological activities; it is also important to evaluate the treating efficacy of the novel oral formulation in the wet age-related macular degeneration (WAMD). Few drugs have been reported to pass through blood-retinal barrier (BRB) by oral administration. In our results, the oral administration of YSB201 enabled the delivery of a therapeutically effective amount of UDCA to the eyes through BRB, resulting in a strong suppression of choroidal neovascularization in the WAMD animal model.

Before analyzing the biological samples of the test group treated with the tested agent, the HPLC analysis method for the test analysis of the sample was first verified, and the sample analysis was performed under the consistent analysis condition. In addition to UDCA, the main component of the test product YSB201, metabolites produced by drug metabolism enzymes, etc., should be quantitatively analyzed. Therefore, we verified that bile acid standards could be evaluated by HPLC in the same assay environment. A high linearity calibration curve was obtained by quantitative determination of standardized bile acid substances (15 total substances) with high purity with a HPLC fluorescence detector using an enzyme reaction column. This calibration curve was used to calculate the concentration of extracted bile components in each blood and organ [29].

Following oral administration of the formulation, the plasma level of UDCA rapidly increased, and the average maximal concentration reached 38.18 μg/mL within 5 min after the dose. Since the elimination of UDCA occurred in a short time, the concentration rapidly decreased, and half of it was out of blood within 1 h after oral administration of drug solution. Rapid absorption, distribution to systemic circulation and eyes, and elimination from the body were the characteristics of UDCA-containing formulation, YSB201, after oral administration in mice models. Based on our observations, the maximal concentration of UDCA in plasma by oral administration of YSB201 was much higher than the concentration of UDCA by oral administration reported in previous research [30,31,32]. The main reason underlying this higher plasma concentration after oral administration might be related to the aqueous solubility of UDCA in the YSB201 formulation and stability in pH range in the solution, resulting in maintenance of aqueous state with the least drug precipitation even under all the pH conditions of the human gastrointestinal tract.

After oral administration of YSB201 (125 mg/kg) as a single dose, the bile acid concentrations were analyzed in plasma or eye samples obtained from the test group mice (*n* = 3) at each time point (5 min–48 h). In the further experiment, bile acid concentration data of control mice (*n* = 3), which were fasted and fed with the same schedule as the test group mice, were obtained from mice plasma and eyes. The experimental results of the test group mice were calculated by reflecting the control mice (*n* = 3) data. By removing the amount of endogenous bile acids from the levels of UDCA and other bile acid substances distributed in the plasma and eyes of the test group mice at each time point after oral administration of the YSB201 preparation, we analyzed the amount of bile acids directly associated with orally administered UDCA concentration trends. Overall, the concentrations of UDCA-type bile acids (i.e., UDCA and TUDCA) in plasma and eyes were higher than the combined amounts of other types of bile acids (Figure 7).

After oral administration of the novel UDCA formulation, mice retinal function and choroidal angiogenesis levels in the test group (YSB201 125 mg/kg/day or 250 mg/kg/day after a laser injury) were compared to those of the negative control mice group (neither laser injury, nor drug treatment) and the vehicle group (treated with vehicle solution after a laser injury). The results of OCT or FFA imaging and western blot analysis showed that the neovascularization in mice choroids were inhibited by YSB201 treatment dosed at 125 mg/kg/day with the reduced expression of VEGF, as compared to the vehicle group (Figure 8). The serial treatment of YSB201 at 125 mg/kg/day for 10 days after the laser injury prevented the angiogenesis to return to the normal choroidal tissue condition as shown in the normal group case (Figure 8). A laser injury caused the malfunctioned retina in the ERG diagram (normal vs. vehicle group) (Figure 9). YSB201 dosed at 125 mg/kg/day led to the recovery of retinal function after laser injury. However, with a higher dose of YSB201 at 250 mg/kg/day, the recovery effects in retinal function were not apparently higher than in the case of YSB201 at 125 mg/kg/day. As indicated by the results shown in Figure 8, YSB201 at 250 mg/kg/day also showed lower treatment effects than YSB201 at 125 mg/kg/day after oral administration. This outcome might be related to decreasing antioxidant effects with the higher dose than the optimal drug dose [33,34]. Kendall et al. showed dose dependency of neuroprotective effects of TUDCA in the mice model of retinal degeneration, resulting in increasing antioxidant effects as more drug doses were administered [33]. However, when Balb/C mice were intravitreally injected with a dose higher than 5 μg of TUDCA (5 mg/mL), A- and B-wave amplitudes in the ERG and outer nuclear layer thickness were reduced, which could be regarded as an increased signal of apoptosis. According to our results, with the dose of 125 mg/kg, UDCA and the major metabolite, TUDCA, are potent treatment agents for preventing the choroidal neovascularization, and those molecules were successfully transported through the blood retinal barriers after the oral delivery.

## 5. Conclusions

In conclusion, to the best of our knowledge, the present study is the first report about the pharmacokinetics and WAMD efficacy profiling experiments of oral UDCA formulation with high water solubility in mice. This novel formulation resolved the limitations of UDCA in physical stability in the aqueous solution with neutral pH range and oral bioavailability. Furthermore, we also demonstrated the applicable feasibility of UDCA as the potent treatment agents in the ocular complications, such as the wet aged macular degeneration (WAMD). The results of this preclinical study may contribute to guiding clinical uses of UDCA as a potential agent to prevent or treat retinal implications including WAMD.

## Figures and Tables

**Table 1:** HPLC method validation results of glycine-conjugated bile acids (G-BA) in mice plasma and eyes (*n* = 3) ^1^.

Bile Acid (Peak No.)	Plasma			Eyes		
	Conc. (μM)	Precision (%)	Accuracy (%)	Conc. (μM)	Precision (%)	Accuracy (%)
**G-BA**	0.2	10.07	98 (9.32)	0.2	7.08	113 (3.60)
	0.5	4.18	101 (5.34)	0.5	9.43	100 (5.96)
GUDCA (**1**)	1	9.07	95 (8.32)	1	1.07	100 (1.30)
	10	4.66	107 (3.37)	10	6.28	113 (3.04)
	100	5.25	101 (1.33)	100	3.47	99 (0.97)
GCA (**4**)	0.2	9.54	107 (10.56)	0.2	7.15	109 (2.30)
	0.5	4.62	98 (5.57)	0.5	13.02	99 (9.73)
	1	11.36	97 (8.70)	1	2.53	100 (2.19)
	10	5.50	104 (2.60)	10	7.21	110 (3.62)
	100	3.85	101 (1.34)	100	2.90	99 (0.77)
GCDCA (**7**)	0.2	12.09	100 (11.12)	0.2	26.02	117 (4.47)
	0.5	4.34	98 (3.85)	0.5	19.75	101 (6.13)
	1	9.51	95 (9.50)	1	8.70	99 (1.59)
	10	4.72	104 (3.46)	10	10.66	108 (6.35)
	100	4.24	101 (1.34)	100	5.65	100 (3.34)
GDCA (**9**)	0.2	11.48	103 (9.64)	0.2	28.89	122 (12.38)
	0.5	5.76	99 (7.20)	0.5	19.63	102 (7.14)
	1	8.91	97 (8.60)	1	7.14	99 (1.78)
	10	4.48	104 (3.26)	10	11.01	108 (7.30)
	100	4.98	101 (1.41)	100	5.70	99 (1.45)
GLCA (**13**)	0.2	13.18	107 (5.31)	0.1	27.41	103 (16.17)
	0.5	7.00	99 (5.70)	0.2	9.12	101 (7.66)
	1	8.03	94 (8.29)	1	9.36	101 (1.04)
	10	4.79	104 (3.03)	10	9.12	109 (5.75)
	100	5.01	101 (1.36)	100	2.82	100 (0.24)

^1^ RSD (residual deviation, %) values are displayed in the parenthesis for the averaged accuracy (%) values.

**Table 2:** HPLC method validation results of taurine-conjugated bile acids (T-BA) in mice plasma and eyes (*n* = 3) ^1^.

Bile Acid (Peak No.)	Plasma			Eyes		
	Conc. (μM)	Precision (%)	Accuracy (%)	Conc. (μM)	Precision (%)	Accuracy (%)
**T-BA**	0.2	6.48	99 (10.06)	0.2	13.64	110 (1.91)
	0.5	3.41	100 (4.58)	0.5	5.05	100 (1.39)
TUDCA (**2**)	1	8.58	96 (7.57)	1	0.91	100 (0.42)
	10	3.76	104 (2.47)	10	6.31	113 (3.08)
	100	4.61	101 (1.15)	100	2.89	99 (0.90)
TCA (**5**)	0.2	12.47	103 (9.08)	0.2	3.77	110 (7.98)
	0.5	3.50	99 (6.50)	0.5	5.70	101 (6.39)
	1	10.72	97 (8.16)	1	3.56	99 (1.50)
	10	5.28	101 (2.96)	10	7.33	102 (14.04)
	100	2.47	101 (1.07)	100	2.81	99 (0.54)
TCDCA (**8**)	0.2	9.40	104 (10.79)	0.05	25.51	79 (52.07)
	0.5	4.06	99 (6.19)	0.1	16.11	89 (31.35)
	1	10.64	97 (8.42)	1	7.06	100 (1.76)
	10	5.08	105 (4.04)	10	10.91	109 (6.73)
	100	3.12	103 (5.52)	100	6.09	99 (1.50)
TDCA (**10**)	0.2	8.95	105 (10.59)	0.1	46.04	93 (8.18)
	0.5	3.34	102 (3.96)	0.5	17.85	101 (9.19)
	1	8.10	97 (7.04)	1	7.42	99 (2.41)
	10	4.44	103 (3.44)	10	11.23	109 (7.43)
	100	4.22	101 (1.32)	100	5.73	99 (1.48)
TLCA (**14**)	0.2	15.54	98 (10.56)	0.2	14.01	101 (9.39)
	0.5	7.80	99 (2.88)	-	-	-
	1	13.40	100 (7.57)	1	3.52	100 (0.46)
	10	4.38	103 (3.28)	10	7.07	107 (3.29)
	100	3.64	102 (1.14)	100	2.04	100 (0.15)

^1^ RSD (residual deviation, %) values are displayed in the parenthesis for the averaged accuracy (%) values.

**Table 3:** HPLC method validation results of unconjugated bile acids (u-BA) in mice plasma and eyes (*n* = 3) ^1^.

Bile Acid (Peak No.)	Plasma			Eyes		
	Conc. (μM)	Precision (%)	Accuracy (%)	Conc. (μM)	Precision (%)	Accuracy (%)
**u-BA**	0.2	9.01	102 (10.82)	0.2	36.77	104 (20.36)
	0.5	4.55	99 (4.57)	0.5	7.24	99 (4.74)
UDCA (**3**)	1	8.58	96 (7.31)	2	14.56	108 (19.22)
	10	4.26	106 (2.80)	10	6.67	114 (2.77)
	100	5.08	101 (1.37)	100	2.70	99 (0.91)
CA (**6**)	0.2	7.19	96 (13.40)	0.1	22.42	89 (13.90)
	0.5	4.89	102 (3.64)	0.5	15.18	104 (12.17)
	1	8.67	98 (7.43)	1	4.62	99 (2.99)
	10	5.32	103 (2.90)	10	7.73	108 (4.22)
	100	2.27	101 (1.20)	100	3.14	99 (0.79)
CDCA (**11**)	0.2	23.81	101 (10.02)	0.1	31.96	90 (6.72)
	0.5	5.66	101 (7.62)	0.5	16.60	96 (7.28)
	1	9.77	95 (7.66)	1	7.46	101 (1.25)
	10	5.39	103 (2.86)	10	12.03	109 (8.10)
	100	3.05	101 (1.25)	100	4.93	99 (1.27)
DCA (**12**)	0.2	5.73	104 (10.53)	0.1	27.87	102 (13.76)
	0.5	4.25	100 (5.18)	0.5	19.08	96 (5.35)
	1	8.22	96 (8.06)	1	5.91	101 (1.07)
	10	4.74	103 (3.07)	10	11.26	111 (7.34)
	100	4.28	101 (1.23)	100	4.51	99 (1.01)
LCA (**16**)	0.2	17.75	99 (9.72)	0.2	21.92	86 (8.31)
	0.5	12.08	102 (11.57)	0.5	22.05	101 (12.31)
	1	12.07	98 (12.86)	1	3.35	100 (2.75)
	10	4.66	102 (2.89)	10	9.60	105 (6.72)
	100	4.02	101 (1.17)	100	4.81	100 (1.51)

^1^ RSD (residual deviation, %) values are displayed in the parenthesis for the averaged accuracy (%) values.

**Table 4:** The pharmacokinetic parameter values of mice plasma and eyes after oral administration of YSB201 (125 mg/kg) (*n* = 3).

PK Parameter (units)	Plasma	Eyes
C_max_ (μg/mL or μg/g of tissues)	38.18 ± 7.05	10.15 ± 7.34
T_max_ (h)	0.08 ± 0.0	0.11 ± 0.05
AUC_(0–48h)_ (h·μg/mL or h·μg/g of tissues)	6.19 ± 1.08	2.14 ± 0.82
k (h^−1^)	0.63 ± 0.09	18.14 ± 0.54
t_1/2_ (h)	1.12 ± 0.15	0.04 ± 0.001
MRT (h)	1.29 ± 0.18	0.27 ± 0.12
